# *WB1*, a Regulator of Endosperm Development in Rice, Is Identified by a Modified MutMap Method

**DOI:** 10.3390/ijms19082159

**Published:** 2018-07-24

**Authors:** Hong Wang, Yingxin Zhang, Lianping Sun, Peng Xu, Ranran Tu, Shuai Meng, Weixun Wu, Galal Bakr Anis, Kashif Hussain, Aamiar Riaz, Daibo Chen, Liyong Cao, Shihua Cheng, Xihong Shen

**Affiliations:** 1 Key Laboratory for Zhejiang Super Rice Research, State Key Laboratory of Rice Biology, China National Rice Research Institute, Hangzhou 311400, Zhejiang, China; wjiyinh@126.com (H.W.); zyxrice@163.com (Y.Z.); slphongjun8868@126.com (L.S.); cnrri_pengxu@163.com (P.X.); 18883948050@163.com (R.T.); mengrice@163.com (S.M.); wuweixun@caas.cn (W.W.); galalanis5@gmail.com (G.B.A.); king3231251@gmail.com (K.H.); aamirriaz33@gmail.com (A.R.); cdb840925@163.com (D.C.); 2 Rice Research and Training Center, Field Crops Research Institute, Agriculture Research Center, Kafr Elsheikh 33717, Egypt

**Keywords:** *Oryza sativa*, endosperm development, rice quality, *WB1*, the modified MutMap method

## Abstract

Abnormally developed endosperm strongly affects rice (*Oryza sativa*) appearance quality and grain weight. Endosperm formation is a complex process, and although many enzymes and related regulators have been identified, many other related factors remain largely unknown. Here, we report the isolation and characterization of a recessive mutation of *White Belly 1* (*WB1*), which regulates rice endosperm development, using a modified MutMap method in the rice mutant *wb1*. The *wb1* mutant develops a white-belly endosperm and abnormal starch granules in the inner portion of white grains. Representative of the white-belly phenotype, grains of *wb1* showed a higher grain chalkiness rate and degree and a lower 1000-grain weight (decreased by ~34%), in comparison with that of Wild Type (WT). The contents of amylose and amylopectin in *wb1* significantly decreased, and its physical properties were also altered. We adopted the modified MutMap method to identify 2.52 Mb candidate regions with a high specificity, where we detected 275 SNPs in chromosome 4. Finally, we identified 19 SNPs at 12 candidate genes. Transcript levels analysis of all candidate genes showed that *WB1* (*Os04t0413500*), encoding a cell-wall invertase, was the most probable cause of white-belly endosperm phenotype. Switching off *WB1* with the CRISPR/cas9 system in Japonica cv. Nipponbare demonstrates that *WB1* regulates endosperm development and that different mutations of *WB1* disrupt its biological function. All of these results taken together suggest that the *wb1* mutant is controlled by the mutation of *WB1*, and that the modified MutMap method is feasible to identify mutant genes, and could promote genetic improvement in rice.

## 1. Introduction

Rice (*Oryza sativa*), one of the most important food crops in the world, provides more than 21% of human caloric needs [1]. With the improvement of living standards, there is increasing demand for high-quality rice, with greater quality of exterior, eating, and processing. Quality of rice appearance and yield are negatively affected by abnormally developed endosperm, which leads to grains with decreased weight and floury endosperm [2,3,4,5,6,7,8], shrunken endosperm [9,10,11,12,13], and great chalkiness [14,15]. Therefore, elucidating the mechanisms of endosperm development will be conducive to cultivating rice varieties with better appearance and higher yield.

Previous studies have shown that abnormality of rice endosperm can be caused by disorder of starch biosynthesis in the endosperm. Starch in the endosperm is composed of amylopectin (α-1,6-branched polyglucan) and amylose (α-1,4-polyglucan) [16]. In recent years, many key genes involved in starch biosynthesis have been identified in rice endosperm. The primary substrate of starch biosynthesis in rice endosperm comes from sucrose in the cell during photosynthesis [17], and it must be transported to the endosperm before being converted to glucose and fructose utilized for starch synthesis [18]. Several key genes involved in this process have been identified, including *OsSUT2*, which encodes a sucrose transporter and plays a vital role in transporting sucrose from source to sinks [19], *GIF1*, which encodes cell-wall invertase and is essential for the hydrolysis and uploading of sucrose [14], *OsSWEET4*, which encodes a hexose transporter and enhances sugar import into the endosperm from maternal phloem [20], and some genes (*SUS2*, *SUS3*, *SUS4*) from the sucrose synthase (SUS) genes family, which play an important role in the hydrolysis of sucrose [21]. However, glucose and fructose are not the direct substrate for starch synthesis: both need to be further converted to glucose 1-phosphate (G1P) under the catalysis of a series of enzymes [18]. The reaction of G1P with ATP (Adenosine 5′-triphosphate) produces the activated glucosyl donor ADP (Adenosine diphosphate)-glucose (ADPG), which is catalyzed by the enzyme ADP-glucose pyrophosphoryase (AGPase). In rice, the AGP gene family is made up of six subunit genes: two small subunit genes, *OsAGPS1* and *OsAGPS2* (*OsAGPS2a*, *OsAGPS2b*), and four large subunit genes, *OsAGPL1*, *OsAGPL2*, *OsAGPL3* and *OsAGPL4*. *OsAGPS1*, *OsAGPS2b*, *OsAGPL1* and *OsAGPL2* mainly function in rice endosperm [9,11,22]. In addition, pyruvate orthophosphate dikinase (PPDK) which is encoded by *OsPPDKB*, is involved in activating fructose [23]. In rice, loss-of-function mutants of these genes show abnormally developed endosperm, thus causing negative impacts on rice appearance quality and grain weight.

The activated substrates must cross the membrane of the amyloplasts before amylose and amylopectin are synthesized in the amyloplasts of the endosperm cells. During this transportation from cytoplasm to amyloplast, the major ADP-glucose transporter encoded by the *Brittle1* (*BT1*) imports the ADPG into amyloplasts; mutants with a defect in *BT1* develop shrunken endosperm [12,13]. When the activated substrates have been transported from the cytoplasm to the amyloplast, the gene *Waxy* encodes granule-bound starch synthase I (GBSS I), which primarily controls amylose synthesis [24]. Other genes control amylopectin synthesis in rice endosperm, including *SSI* [25], *SSIIa* [26], and *OsSSIIIa* [27], which encode starch synthase, *ISA1* [28,29] encoding isoamylase-type DBE isoamylase 1, and *OsBEIIb* [30] encoding starch branching enzymeIIb. Loss-of-function mutants of these genes severely disrupt the normal development of the endosperm.

Some regulators involved in starch synthesis during endosperm development have also been identified. *FLO2* mediates a protein-protein interaction, with a mutation of *FLO2* resulting in a floury endosperm [2]. *FLO6* directly interacts with *ISA1*, which affects the formation of starch granules during development of the rice endosperm [4]. *FLO7* encodes a regulator involved in starch synthesis and amylopectin development of the peripheral endosperm [8]. *Rice Starch Regulator1* (*RSR1*), a member of the AP2/EREBP family of transcription factors, negatively regulates starch synthesis [31]. *SUBSTANDARD STARCH GRAIN4* (*SSG4*) regulates the size of starch grains (SGs) in rice endosperm [6].

Deformity of the endosperm can also result from dysregulation of development of the protein bodies and storage proteins in the rice endosperm. *Chalk5*, which encodes a vacuolar H^+^-translocating pyrophosphatase with inorganic pyrophosphate hydrolysis and H^+^-translocation activity, is a major quantitative trait locus (QTL) which controls grain chalkiness [15]. The rice basic leucine Zipper factor (RISBZ1) and rice prolamin box binding factor (RPBF) are transcriptional activators, which coordinate to regulate the expression of SSP (seed storage protein) genes [32]. Decreased expression of *RISBZ1* (*OsbZIP58*) and *RPBF* in transgenic plants causes opaque endosperm.

**Figure 1:**
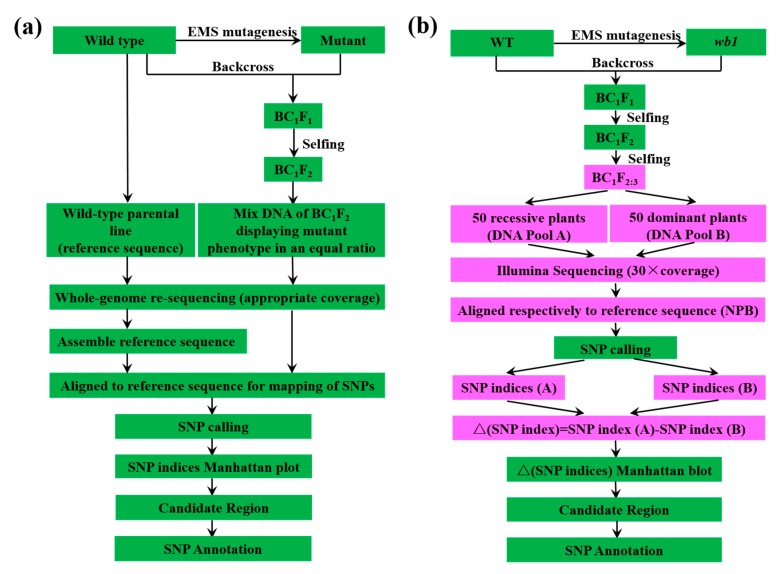
The steps of MutMap method applied to rice. (**a**) Common scheme of MutMap method applied to rice following the protocol described as previously reported [33]; (**b**) The scheme of gene mapping used in this study. The BC_1_F_2:3_ progeny formed mapping population. DNA of 50 recessive and 50 dominant plants from mapping population are mixed separately in an equal ratio to form the DNA Pool (A) and Pool (B) followed by the construction of DNA library and Illumina sequencing with 30×coverage, and then the treated sequencing data were aligned with the reference sequence followed by single nucleotide polymorphisms (SNP) calling. The reference sequence is the publicly available Nipponbare rice genome sequence [37]. For each identified SNP, SNP index (A) was obtained from Pool (A) and SNP index (B) corresponds with Pool (B). SNP index (A) minus SNP index (B) is ∆ (SNP index) which is used for Manhattan plot, and we can obtain candidate region followed by SNP annotation. The pink color represents the different steps compared to the common scheme of MutMap. EMS: Ethane methyl sulfonate.

Several methods are currently used for gene isolation. The most commonly used one is positional cloning (map-based cloning), by which many rice genes were isolated. However, map-based cloning is more time- and labor-intensive for isolating genes, especially QTLs. Therefore, many researchers have been exploring new methods for genes isolation. MutMap (Figure 1a) [33], based on next-generation sequencing (NGS) [34], is a recently developed method of rapid gene isolation. The MutMap method has been used to isolate some rice genes, including *OsRR22*, a gene responsible for the salinity-tolerant phenotype of *hst1* [35] and *Pii*, a gene enhancing rice blast resistance [36].

Although the understanding of the molecular mechanisms of the formation of rice endosperm has made great progress, rice endosperm is a very complex agronomic trait. Hence, it is still necessary to identify more functional genes and to describe their molecular mechanisms in order to enable systematic and comprehensive understanding of the inheritance of rice endosperm formation. In this study, we isolated *WB1*, which controlled rice endosperm development, via the modified MutMap method, and found that *WB1* played an important role in the regulation of starch synthesis during rice endosperm development. We also verified the target gene by CRISPR/Cas9 system. Our study also played a crucial role in explaining the molecular mechanisms of the formation of rice endosperm and the exploration of new methods for gene mapping.

## 2. Results

### 2.1. Phenotypic Characterization and Genetic Analysis of the wb1 Mutant

**Figure 2:**
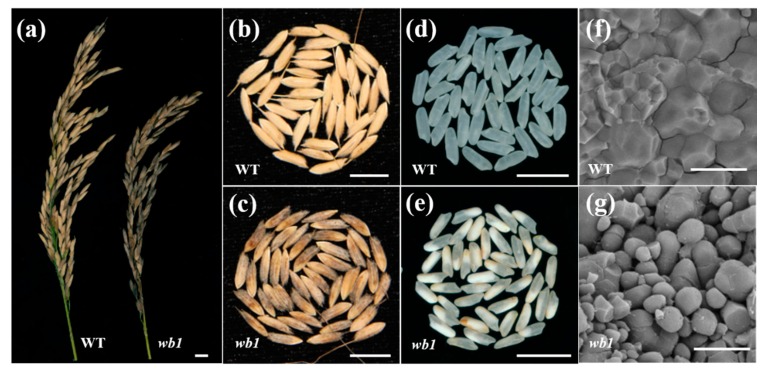

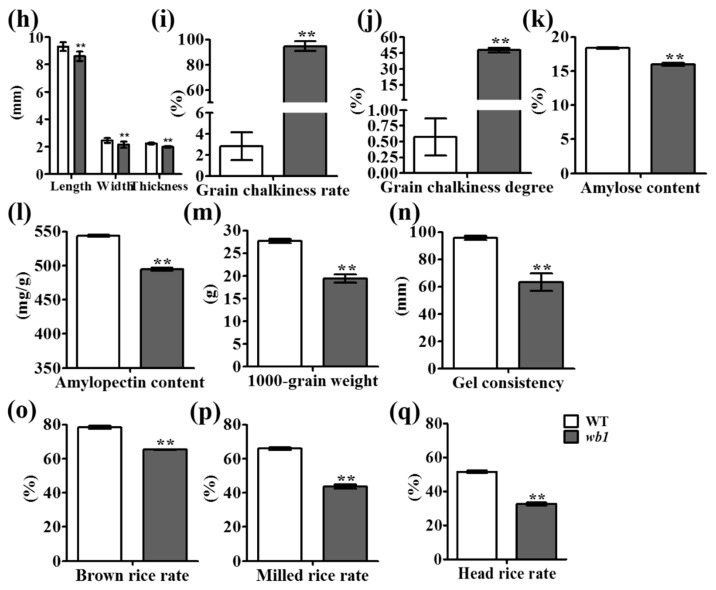
Phenotypic analyses of *wb1*. (**a**) Comparison of representative WT and *wb1* plant panicles; (**b**,**c**) Appearance of WT (**b**) and *wb1* (**c**) grains; (**d**,**e**) Appearance of WT (**d**) and *wb1* (**e**) white grains; (**f**,**g**) Scanning electron microscope images of WT (**f**) and *wb1* (**g**) seed endosperm. Magnification, ×2000; (**h**) Measurements of seed length, width, and thickness of WT and *wb1* grains (*n* = 20); (**i**) Grain chalkiness rate comparison of WT and *wb1* grains (*n* = 6), and grain chalkiness rate is the rate of chalky grains in total grains; (**j**) Grain chalkiness degree comparison of WT and *wb1* grains (*n* = 6), and grain chalkiness degree is the grain chalkiness rate multiplied by grain chalkiness area (the percent area of chalk in a grain); (**k**) Amylose content comparison of WT and *wb1* grains (0.05 g grain powder each, *n* = 3); (**l**) Amylopectin content comparison of WT and *wb1* grains (0.01 g grain powder each, *n* = 3); (**m**) Comparison of 1000-grain weight of WT and *wb1* (*n* = 10); (**n**) Gel consistency comparison of WT and *wb1* grains (*n* = 4); (**o**,**p**,**q**) Comparisons of brown rice, milled rice and head rice rates of WT and *wb1* (25 g paddy each, *n* = 3). Data are given as means ± SD (standard deviation). The asterisks represent statistical significance between WT and *wb1*, determined by a student’s *t*-test (** *p* ≤ 0.01). Scale bars: (**a**–**e**) 10 mm; (**f**,**g**) 30 μm.

To identify new regulators of endosperm development, we recovered an endosperm development defective mutant named *wb1* from a mutant pool (in the *Japonica* variety ChangLiGeng background). The *wb1* mutant showed no apparent differences from WT throughout the vegetative stage. Plant height and the number of panicles per plant of *wb1* plants were similar to those of the WT at the mature stage. The number of spikelets per panicle, number of grains per panicle, seed-setting rate and 1000-grain weight of *wb1* were all showed a marked decreased compared with those of the WT (Table S1). Unlike WT, the glume of *wb1* grains showed brown color (Figure 2a–c), and *wb1* displayed markedly more grain chalkiness in the grain belly (Figure 2d,e). Grain chalkiness rate and grain chalkiness degree were 94.8% and 47.6% in *wb1* grains, while those of WT grains were 2.8% and 0.6% (Figure 2i,j). Scanning electron microscope images clearly indicated that the endosperm from grains of *wb1* developed abnormally as a result of loosely packed, spherical starch granules, in contrast to the densely packed, irregularly polyhedral starch granules of the normal endosperm from the grains of WT (Figure 2f,g). Grain size measurements showed that the seed length, width, and thickness were all significantly reduced in *wb1* grains (Figure 2h), resulting in a smaller grain size than that of WT, even occasionally in a shriveled phenotype. We also measured amylose and amylopectin content of the mature grains of *wb1* and WT. Amylose and amylopectin content were remarkably decreased in *wb1* grains (Figure 2k,l), suggesting that the starch accumulation in *wb1* grains was severely disrupted. All these results collectively reveal that mutation of *WB1* caused a defect in the endosperm development, which led to the higher grain chalkiness degree and a significant reduction of 1000-grain weight in *wb1* grains. We also analyzed physical properties of *wb1* and WT grains, including gel consistency (Figure 2n), brown rice rate (Figure 2o), milled rice rate (Figure 2p) and head rice rate (Figure 2q). Each of these was significantly reduced in the mutant, suggesting that dramatic physical changes have occurred in the *wb1* grains, which will further affect rice processing and eating quality.

To verify that this locus associated with the *wb1* phenotype was controlled by a single recessive gene, genetic analysis was conducted to examine the phenotype of all plants from BC_1_F_1_ and F_1_ progeny, and of 1087 and 1000 plants from BC_1_F_2_ and F_2_ progeny, respectively. The results showed that BC_1_F_1_ and F_1_ seeds exhibited the wild-type phenotype, while the segregation model of normal to chalky grains fitted well to the expected ratio of a single inheritance, 3:1 (820:267, 745:255), in the BC_1_F_2_ and F_2_ progeny (Table S2).

### 2.2. Candidate Region of the WB1 Gene Obtained through the Modified MutMap Method

**Figure 3:**
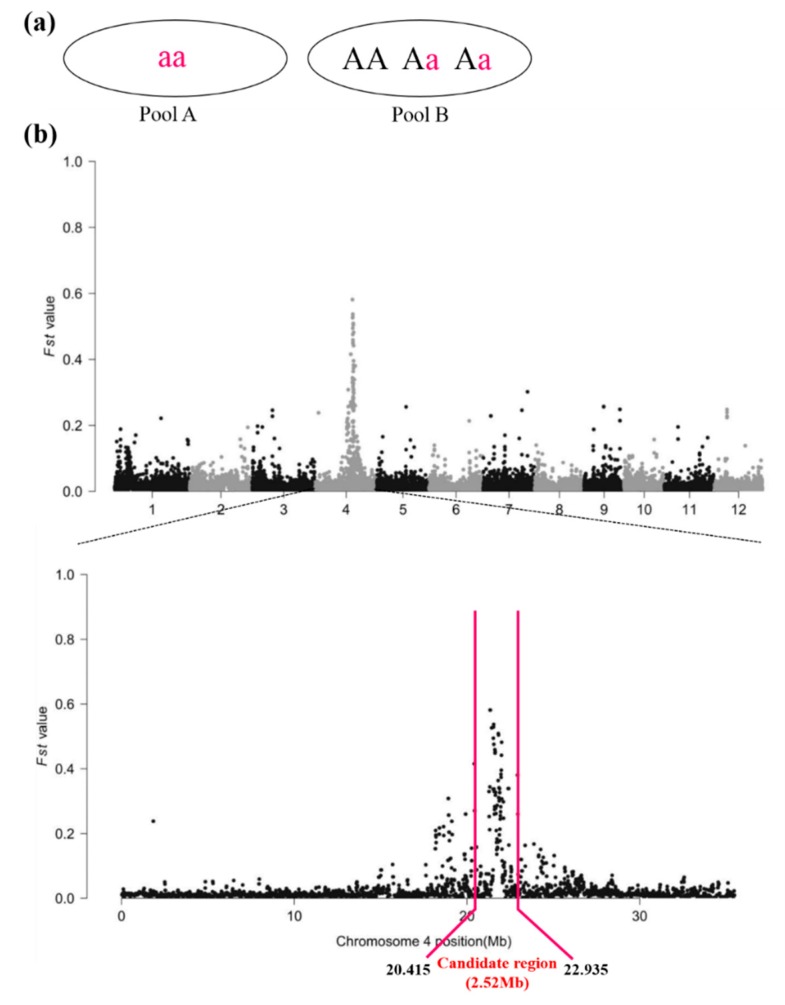
Candidate region of wb1 obtained by the modified MutMap method. (**a**) An example for explanation of ∆ (SNP index) for the casual SNP. Theoretically, SNP index (A) would be 1, SNP index (B) 0.333 (1/3), and thus ∆ (SNP index) would be 0.667 (1 minus 1/3); (**b**) ∆ (SNP indices) Manhattan plot. *Fst* value, defined as the proportion of genetic diversity due to allele frequency differences among populations described by the previous report [38]. ∆ (SNP indices) and *Fst* values have the same meaning in this study.

A modified MutMap method (Figure 1b) was applied to isolate the WB1 gene. After re-sequencing for Pool A and Pool B, we obtained 125,252,285 (SRA accession SRP135580) and 120,484,878 (SRA accession SRP135578) cleaned reads for Pool A and Pool B, respectively, corresponding to >20 Gb of total read length with 30× coverage of the rice genome (370 Mb; Table S3). After these cleaned reads were aligned separately to the Nipponbare reference sequence by the BWA software, we obtained 110,119,455 and 105,488,179 unique mapped reads for Pool A and Pool B, respectively, corresponding to 87.55% and 87.92% coverage of the rice genome (Table S4). Then we calculated ∆ (SNP indices) or *Fst* value based on the sliding window of the whole genome scan following by plotting the ∆ (SNP indices) for all 12 chromosomes of rice (Figure 3b). As we expected, ∆ (SNP indices) were distributed randomly around 0 for most parts of the genome (Figure 3b). Finally, we obtained the candidate region of 2.52 Mb (Figure 3b).

### 2.3. Screening the SNPs Detected in the Candidate Region

From the ∆ (SNP indices) plot (Figure 3b), we obtained the candidate region of 2.52 Mb where we detected 275 SNPs in chromosome 4 followed by gene annotation (Table S5). To identify the true causal SNP, we screened these SNPs with three steps: (i) retaining the SNPs in which ∆ (SNP indices) ranged from 0.6 to 0.8; (ii) removing SNPs located in the intergenic region and SNPs which resulted in synonymous substitutions; and (iii) detecting the SNPs between WT and wb1 by sequencing. As a result, we obtained nineteen SNPs, which were located in twelve candidate genes (Table 1).

### 2.4. Identification of the Casual SNP

**Figure 4:**
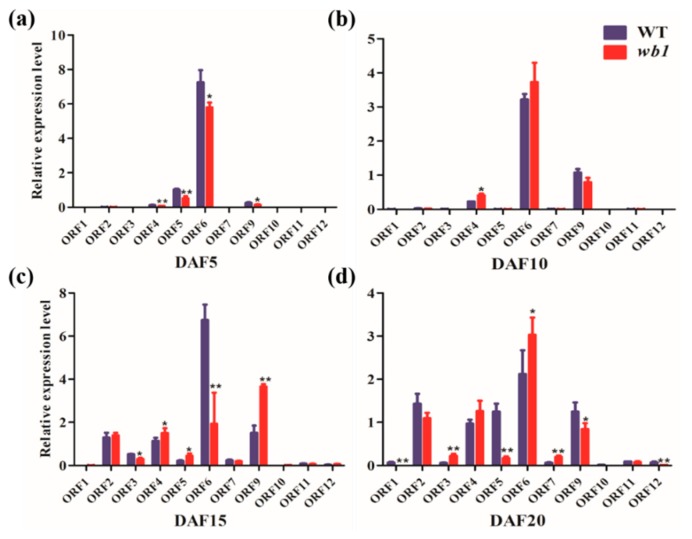
Relative expression analysis of 11 candidate genes based on real-time quantitative PCR (qPCR) at four stages of endosperm development between WT and *wb1*. (**a**) Relative expression analysis of 11 candidate genes at DAF5 stage; (**b**) Relative expression analysis of 11 candidate genes at DAF10 stage; (**c**) Relative expression analysis of 11 candidate genes at DAF15 stage; (**d**) Relative expression analysis of 11 candidate genes at DAF20 stage. All data were compared with transcript levels of WT by Student’s *t*-test (* *p* ≤ 0.05, ** *p* ≤ 0.01). Values were means ± SD (*n* = 3).

We detected the expression levels of twelve candidate genes in endosperm tissues at various development stages (5, 10, 15 and 20 DAF) (Figure 4). We successfully detected all genes transcript levels except for that of *ORF8*. *ORF6* maintained relatively high expression level in comparison with other genes and its transcript level changed significantly during the four stages of endosperm development between WT and *wb1* (Figure 4). Although some genes demonstrated higher transcript levels at the DAF15 and DAF20 stages (Figure 4c,d) compared with the DAF5 and DAF10 stages (Figure 4a,b), and the transcript levels of several genes were also significantly altered between WT and *wb1* (Figure 4), all other genes showed low expression levels on the whole in contrast to the transcript levels of *ORF6*. Therefore, we may conclude that the mutation of *ORF6* (*Os04t0413500* or *Os04g33740*) played a major role in the defect of *wb1*.

**Figure 5:**
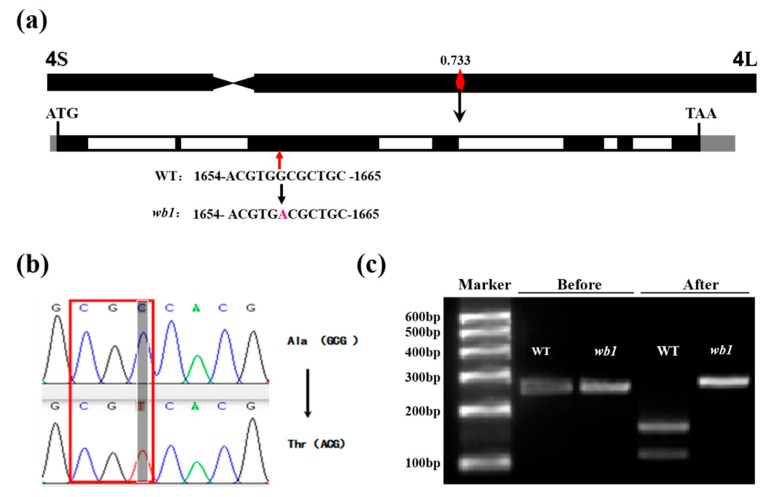
Further verification of causal SNP in *wb1*. (**a**,**b**) Sequencing validation of the causal SNP and the type of mutation; the red arrow indicates the mutant site, and the black arrows indicate the alternations; (**c**) Digestion of restriction endonuclease *Hae*II. “Before” represents the non-treated PCR product and “After” represents the *Hae*II-treated PCR product.

SNP-20423829 were G to A transitions, presumably caused by EMS mutagenesis [39], and it was located at the site 1659 bp of the third exon of *ORF6* encoding a glycosyl hydrolase. This SNP led to an A159T mutation (codon GCG to ACG; Figure 5a,b). Moreover, results of digestion of restriction endonuclease *Hae* II confirmed this mutant site (Figure 5c). Accordingly, we hypothesized that *wb1* was caused by a missense substitution in *ORF6*. We also found that *ORF6* was a novel allele of *GIF1* (*Os04g33740*) which controlled rice grain filling and yield [14].

### 2.5. Function Verification of the WB1 Gene (ORF6) through the CRISPR/Cas9 System in Reverse

**Figure 6:**
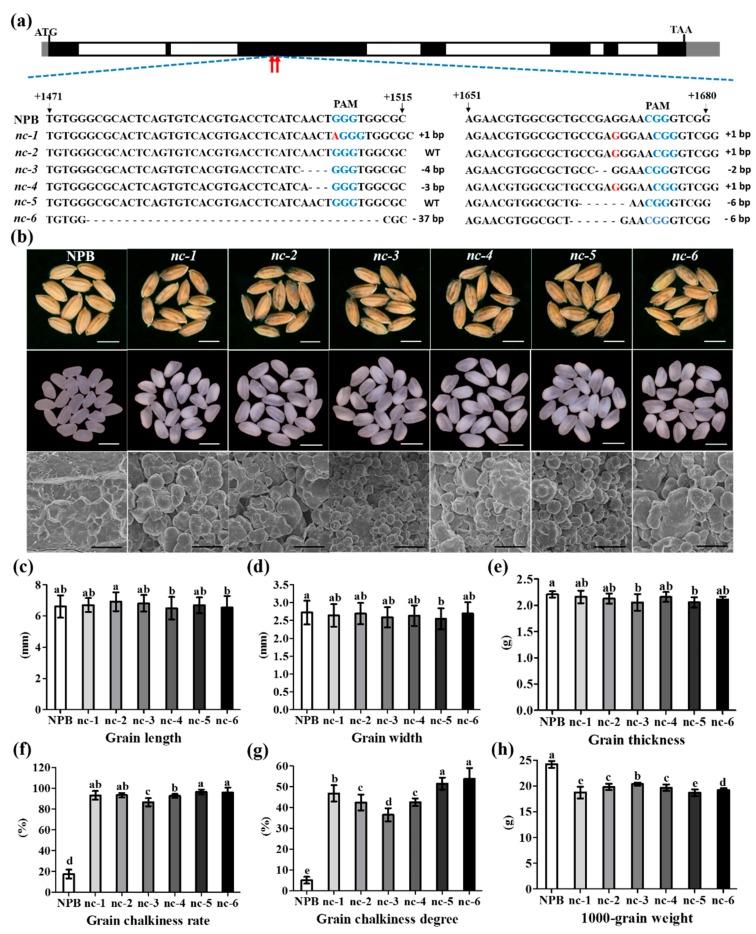
Sequencing validation and phenotypic analyses of six novel allelic mutants. (**a**) Sequencing validation of six novel allelic mutants. Blue color represents the PAM sequence of CRISPR/Cas9 system; red color represents insert bases; “-” represents deletion bases; the red arrows indicate the mutant sites mediated by CRISPR/Cas9 system. (**b**) Appearance and SEM of NPB and mutants grains. Magnification, ×1000; (**c**–**h**) Measurements of grain length (*n* = 30), width (*n* = 30), thickness (*n* = 10), grain chalkiness rate (*n* = 10), grain chalkiness degree (*n* = 10) and 1000-grain weight (*n* = 10) of NPB and mutant lines. Different letters indicate the statistical difference at *p* ≤ 0.05 by Duncan’s test. Values were means ± SD. Scale bars: bars of grains figures 5 mm; bars of SEM figures 10 μm.

To verify our hypothesis, we created six novel alleles of *WB1* through the CRISPR/Cas9 system in *Japonica* cultivar Nipponbare (NPB). We found two target sequences in the third exon of *WB1* corresponding to CRISPR/Cas9 system and obtained six different mutants in T1 lines (Figure 6a). Grains of six mutants displayed brown glumes and grain chalkiness in the grain belly compared with the common grains of NPB (Figure 6b). SEM images distinctly showed that endosperm of six mutants developed abnormally compared to the normal endosperm of NPB (Figure 6b). The phenotypes of six mutants were similar to that of *wb1* (Figure 2b–g). Those results further proved that *WB1* was the target gene responsible for the *wb1* phenotype.

In NPB and six mutant lines, we measured 1000-grain weight (Figure 6h) and the main factors affecting 1000-grain weight, including grain length (Figure 6c), width (Figure 6d), and thickness (Figure 6e), grain chalkiness rate (Figure 6f) and degree (Figure 6g). Duncan’s test indicated that the grain chalkiness rate degree were the major factors causing the significant reduction of 1000-grain weight of six mutant lines. The differences in grain length, width, and thickness between six mutant lines and NPB were not similar to the differences between WT and *wb1* (Figure 2h). This discrepancy was probably caused by the longer grain length of WT (~9.3 mm, Figure 2h) compared with that of NPB (~6.6 mm, Figure 6c) and the different mutations of WB1 between wb1 (Figure 5a) and the six mutant lines (Figure 6a).

**Figure 7:**
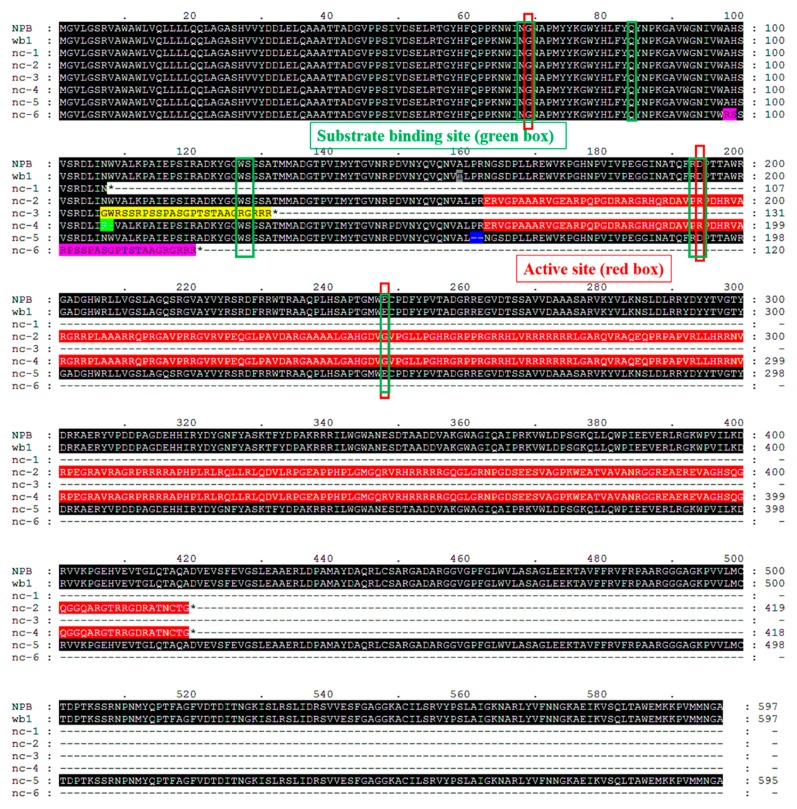
WB1 alignments of NPB (WT) with six mutant lines and *wb1*. Analysis performed with MEGA 5.0 software. The substrate binding site (eight residues, green boxes) and the active site (three residues, red boxes) are indicated by the Blast search program (http://www.ncbi.nlm.nih.gov/BLAST/). Black color indicates a sequence that is consistent with that of WT. Except for the black color, the same color represents the same sequence, and different colors represent the different mutations among the mutant lines.

Interestingly, some differences were also found among the six mutant lines (Figure 6c–h). Those results suggested that the grain chalkiness rate and grain chalkiness degree collectively determined the 1000-grain weight (Figure 6f–h), especially in the mutant line *nc-3*, where grain chalkiness rate and grain chalkiness degree showed significant decreases as compared to the other mutant lines, corresponding to its higher 1000-grain weight. To test whether the differences in the grain chalkiness rate and grain chalkiness degree among the six mutant lines were caused by different mutations of WB1, we performed multiple comparison of WB1 sequences by MEGA 5.0 software (Figure 7). The findings indicated that the six mutant lines showed different mutations which disrupted the substrate binding site and the active site of WB1, except in the mutant line *nc-5* (Figure 7).

### 2.6. Expression Analysis of Starch Metabolism-Related Genes in Endosperm

**Figure 8:**
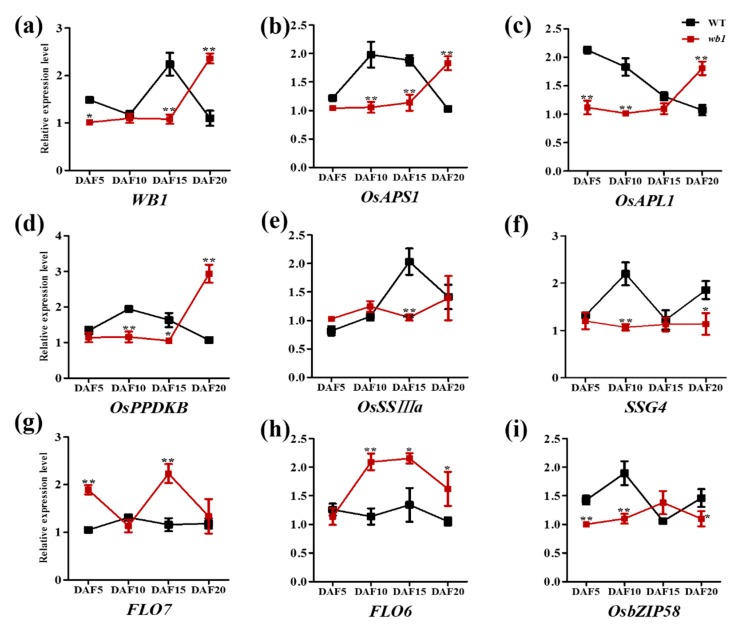
Relative expression analysis of genes associated with starch synthesis by qPCR at four stages of WT and *wb1* endosperm development. (**a**–**i**) Relative expression analysis of *WB1* (**a**), *OsAPS1* (**b**), *OsAPL1* (**c**), *OsPPDKB* (**d**), *OsSSIIIa* (**e**), *SSG4* (**f**), *FLO7* (**g**), *FLO6* (**h**), *OsbZIP58* (**i**) in WT and the wb1 mutant*.* All data were compared with the relative expression levels of WT by Student’s *t*-test (* *p* ≤ 0.05, ** *p* ≤ 0.01). Values were means ± SD (*n* = 3).

We performed qPCR analysis of total RNA extracted from the seed endosperm of WT and *wb1* at various stages (DAF5, DAF10, DAF15, and DAF20) and detected the transcript levels of some genes involved in starch synthesis. As shown in Figure 8, transcript levels of those genes were all altered during development of the rice endosperm. During the critical stages (DAF10 and DAF15) of grain filling, transcript levels of all genes showed a striking contrast between WT and *wb1*. This suggests that altered transcript levels of starch synthesis-related genes are probably involved in the abnormal development of rice endosperm. The higher transcript levels of *WB1*, *OsAPS1*, *OsAPL1*, *OsPPDKB*, and *FLO6* at the mature stage (DAF20) in the *wb1* mutant were probably caused by different maturity of seeds between WT and *wb1*.

## 3. Discussion

### 3.1. WB1 Controls Rice Endosperm Development

Grain chalkiness is one of the most important factors leading to low grain weight [15] and affecting rice appearance and milling, cooking, and eating quality [40,41]. Grain chalkiness is controlled by complex quantitative trait loci and by climatic conditions during rice grain filling, especially high temperature [42]. *Chalk5*, a major quantitative trait locus controlling grain chalkiness, and affecting head rice rate, is the only one that has been identified and characterized up to now. *Chalk5* is involved in the biogenesis of protein bodies in the endosperm cells [15]. Grain chalkiness can be an indicator of abnormally developed endosperm [2,3,4,5,6,7,8]. The major component of rice endosperm is a starch that is mainly composed of amylose and amylopectin. Many genes directly involved in the biosynthesis of amylose and amylopectin in rice endosperm cells have been identified and characterized, such as *Waxy*, *SSI*, *SSIIa*, *OsSSIIIa*, *ISA1*, *OsBEIIb* and *OsPPDKB* [23,24,25,26,27,28,30]. Loss of function of these genes can result in an abnormally developed endosperm, displaying more grain chalkiness and low grain weight.

Sucrose is produced by the source organ or photosynthetic organ and used as a carbon source for starch biosynthesis in endosperm cells. Sucrose must be transported from source organs into sink organs, which occurs via apoplast and/or sympast. Accordingly, in addition to the genes directly involved in starch biosynthesis of endosperm cells, other genes involved in this process can also affect endosperm development. In the apoplastic pathway, sucrose can be converted by cell-wall invertases into glucose and fructose, which are transported into cells by hexose transporters. Sucrose can also be directly taken by sucrose transporters into sink cells where it is hydrolyzed into glucose and fructose by sucrose hydrolases, including SUS2, SUS3, and SUS4 [21]. *GIF1* encodes a cell-wall invertase that mainly functions in the hydrolysis and uploading of sucrose during early grain-filling. The *gif1* mutant shows slower grain filling, ~24% lower final grain weight, and lower contents of amylose and amylopectin, and markedly more grain chalkiness as a result of abnormally developed and loosely packed starch granules [14]. *OsSWEET4* encodes a hexose transporter that is responsible for transferring hexoses across the BETL (basal endosperm transfer layer) to sustain of rice endosperm, downstream of a cell-wall invertase. The *ossweet4-1* mutant shows incomplete grain filling and significantly decreased grain weight [20]. *OsSUT2* encodes a sucrose transporter that functions in sucrose uptake from the vacuole. The *ossut2* mutant has significantly decreased sugar export ability and 1000-grain weight [19]. In our study, *WB1* encoded the *cell-wall invertase 2* (*OsCIN2*) and was a novel allele of the *GIF1* (*Os04t0413500* or *Os04g33740*) gene which controlled rice grain-filling and thus affected rice endosperm development [14]. Due to the *WB1* gene mutation (Figure 5) which led to grain incomplete filling [14], the physical and chemical properties of grain endosperm of the *wb1* mutant have been altered, like higher grain chalkiness rate (Figure 2i), higher grain chalkiness degree (Figure 2j), markedly more grain chalkiness as a result of loosely packed, spherical granules (Figure 2e,g), lower contents of amylose and amylopectin (Figure 2k,l), and ~30.0% lower 1000-grain weight (Figure 2m). In addition, transcript levels of the starch synthesis-related genes in our study varied during rice endosperm development (Figure 8). All of these observations suggest that the *wb1* mutant exhibits a defect in endosperm development, thus leading to the white-belly endosperm with altered phy-chemical property.

### 3.2. Different Mutations of WB1 Can Disrupt Its Biological Function

Many genes make up a large regulatory framework that regulates life activity in higher plants. These genes encode active proteins that are responsible for the major functions in this global regulatory framework. The function of each of active protein is determined by its own primary, secondary, and tertiary structure. In rice, mutations of a gene can result in its encoding protein with structural alterations which can affect its biological function followed by phenotypic changes. The *sd1* gene, well known as the genetic basis for the first “green revolution” in rice, encodes a GA 20-oxidase involved in the GA biosynthesis pathway; the *sd1* gene controls the plant height of rice, and mutations (*sd1-d*, *sd1-r*, *sd1-c*, *sd1-j*) in this locus cause the dwarfism of rice to different degrees [43,44,45].

In our study, six mutants of novel alleles of *WB1* displayed the same phenotype as the *wb1* mutant (Figure 2b–e and Figure 6b), primarily showing higher chalkiness rates (Figure 6f), higher chalkiness degrees (Figure 6g), and lower 1000-grain weight (Figure 6h). The sequence analysis showed that six different mutations have occurred in the WB1 locus (Figure 6a), leading to alterations of the amino acid sequence of the WB1 protein in different types (Figure 7). In the previous research of the *gif1* mutant, the *GIF1* gene revealed a 1-nt deletion in the coding region, causing the premature GIF1 protein which disrupt its biological function (incomplete grain-filling) [14]. The *WB1* gene of *nc-1*, *nc-3* and *nc-6* also caused different premature WB1 proteins with altered substrate binding site and active site (Figure 7). The frame shift mutation of the *WB1* gene of *nc-2* and *nc-4* also disrupted its biological function (Figure 6b and Figure 7). Interestingly, the single amino acid substitution (A159T) of WB1 of *wb1*, and Proline-161 and Arginine-162 deletion of WB1 of *nc-5* led to the dysfunction of WB1 without altered substrate binding site and active site (Figure 2, Figure 6 and Figure 7), suggesting that Alanine-159, Proline-161 and Arginine-162 are required for activity of WB1. Moreover, grain chalkiness degrees and 1000-grain weight showed significant differences among some mutants. However, several mutants showed no differences in grain chalkiness degree and 1000-grain weight (Figure 6g,h); these results suggest that different mutations probably affect the formation of grain chalkiness in different degrees, and further research still needs to be conducted to explain the molecular mechanism. In summary, seven novel alleles (including the *wb1* mutant) had different mutations which disrupted their biological functions.

### 3.3. The Modified MutMap Method Applied to Isolate WB1 Is Feasible for Gene Mapping

MutMap is a new method used for gene identification [33]. Using MutMap method, researchers can isolate mutant genes and QTLs rapidly, accurately, and conveniently compared to conventional map-based cloning [33,35,36]. Through the MutMap method, researchers only sequence the DNA pool from recessive individuals of F_2_ population based on second-generation sequencing, followed by aligning to the assembled whole-genome sequence of wild-type. The population used for the MutMap method is BC_1_F_2_ population, which can show unequivocal segregation between the mutant and wild-type phenotype. Notably, the MutMap method requires assembling the whole-genome sequence of wild type accurately used as the reference sequence.

Previously, many genes have been identified by the MutMap method in rice. *OsRR22*, responsible for the salinity-tolerance phenotype for the *hst1* mutant, has been identified by a MutMap method: the sequence depth and the average coverage of wild-type are 28.7× and 59.9%; the number of BC_1_F_2_ individuals is 20 and the sequence depth is 18.4× [35]. Two mutant genes regarding pale green leaf have been identified: the sequence depth and the average coverage of wild-type are >12× and ~95.5%; the number of BC_1_F_2_ individuals is 20 and the sequence depths are 12.5× and 24.1× [33]. Four mutant genes regarding semi-dwarf phenotype also have been identified: the sequence depth and the average coverage of wild-type are >12× and 82.4%~84.2%; the number of BC_1_F_2_ individuals is 20 and the sequence depths are 14.2×~16.6× [33].

The MutMap method is subject to high error rates because of multiple factors, including difficulty in determining the number of F_2_ progeny showing the mutant phenotype, the average coverage (depth) of genome sequencing, and classification of phenotypes between wild and mutant phenotype [33,46]. Therefore, the greater the number of F_2_ progeny showing the mutant phenotype to be bulked, the deeper the average depth of genome to be sequenced, and more accurate classification of phenotypes between wild and mutant type, the lower the rate of false positives [33].

In our study, a modified MutMap method (Figure 1b) was applied to successfully isolate the *WB1* gene related to endosperm development in rice. Compared with the MutMap method, our modified MutMap method has some advantages. Firstly, the individuals of bulked DNA Pools used for sequencing were from BC_1_F_2:3_, which has a more stable genetic background than the BC_1_F_2_ population; because of the reduced effect from other gene mutations on the target phenotype, it was easier to distinguish plants between mutant type and wild type; Secondly, the appropriately elevated number of BC_1_F_2:3_ individuals (50, Figure 1b) and sequence depth (30×, Figure 1b) ensured relatively high coverage (87.92% and 87.55%; Table S4); Lastly, we sequenced the DNA pools not only from recessive individuals but also from dominant individuals followed by aligning to the reference sequence Nipponbare, respectively; therefore, it was not necessary to sequence and assemble the whole-genome sequence of wild type used as reference sequence. We directly used the Nipponbare genomic sequence as our reference sequence, so that we greatly reduced the costs required for sequencing and assembling reference sequence. Therefore, the *WB1* gene mapping result showed higher specificity with the single peak in the chromosome 4 (Figure 3b) compared to that by the MutMap method [33]. Besides, the modified MutMap method also has a deficiency that is the significant difference in the whole genome sequence between Nipponbare and Wild-type (reference sequence). Delightedly, several rice genome sequences have been published, like *indica* cultivar 9311 [47], Zhenshan97, Minghui63 [48] and Shuhui498 [49] which can be used as reference sequence directly, expanding the application scope of the modified MutMap method. Overall, the modified MutMap method showed a low error rate, a relatively low cost and a high specificity, and could promote the development of rice genetics.

## 4. Materials and Methods

### 4.1. Plant Materials and Growth Condition

The *wb1* (mutant) was initially identified from the ethyl methanesulfonate (EMS)-treated *Japonica* rice variety ChangLiGeng (CLG, Wild Type) M_2_ population. The *wb1*, as the pollen acceptor, was crossed with WT and ZhongHui8015 (ZH8015, *Indica*), respectively. The resulting first filial generation (BC_1_F_1_, F_1_) plant was self-pollinated, and the second generation (BC_1_F_2_, F_2_) was used as the genetic analysis population. We collected seeds of 100 individuals from BC_1_F_2_ population, and then cropped the seeds to obtain 100 pedigrees (BC_1_F_2:3_) which were used as the mapping population for the modified MutMap method (Figure 1b). All plants were grown in an experimental paddy field at China National Rice Research Institute (Hangzhou, Zhejiang province and Lingshui, Hainan province, in China) under natural open-air condition.

### 4.2. Grain Quality Analysis

Scanning electron microscopy was performed as described previously [50]. Measurements of amylose content of mature grains (0.05 g powder) were conducted by HPSEC-MALLS-RI following the method of Fujita et al. (2003) [51]. Quantitative amylopectin content was determined by processing 0.01 g powder of the mature grain, according to a method from a previous report [52]. Each measurement was repeated three times (*n* = 3).

The paddy rice of WT and wb1 were dried to moisture content of 12–14% and were maintained at room temperature at least three months before measuring the brown rice rate, milled rice rate, and head rice rate by grain polisher AH001151 (KETT, Tokyo, Japan), performed as Zhou et al. (2015) [53]. Each measurement of 25 g paddy rice was performed with three replicates (*n* = 3, total 75 g paddy rice). Grain chalkiness rate and grain chalkiness degree were determined using SC-E Rice Quality Inspection and Analysis System (WanShen, Hangzhou, China). The white grains from 12 plants (6 plants from WT and wb1, respectively, *n* = 6) were used for grain chalkiness rate and degree measurements. Gel consistency was measured (*n* = 4) following the protocol described in Li et al. (2014) [15].

### 4.3. PCR, RNA Isolation and Real-time Quantitative PCR (qPCR)

PCR amplifications of candidate genes were performed using KOD FX DNA Polymerase (TOYOBO). The PCR product of the reaction of restriction enzyme *HaeII* digestion was amplified by KOD-Plus-Neo (TOYOBO). The primer pairs designed for this study are listed in Table S6.

Total RNA was prepared from grains of WT and *wb1* at 5, 10, 15, and 20 DAF (days after flowering) using the TIANGEN RNAprep Pure Plant Kit (Tiangen Biotech, Beijing, China). The first cDNA strand was synthesized from DNase I-treated RNA using Oligo-dT (18) primers in a 20 µL reaction system based on a SuperScriptIII Reverse Transcriptase Kit (TOYOBO). qPCR was performed on a Roche Light Cycle 480 device using THUNDERBIRD SYBR qPCR Mix (TOYOBO). Each reaction was performed with three replicates (*n* = 3). The primers used in this analysis are listed in Table S6.

### 4.4. DNA Template Preparation, DNA Library Construction, and Re-Sequencing

Genomic DNA was extracted (large scale) from young leaf tissues following the modified hexadecyl trimethylammonium bromide (CTAB) method [54]. Young leaves (total 5 g, 0.1 g per plant) were obtained from 50 plants displaying the mutant or wild phenotype in the BC_1_F_2:3_ population and were used to prepare the pooled genomic DNA (Pool A and Pool B, respectively) which was used for illumina sequencing. The DNA concentration was measured by Nanodrop 2000 spectrophotometer. ~1 µg, each for both Pool A and Pool B, of total high-quality pooled DNA samples (1.8 ≤ OD260:OD280 ≤ 2.0) was used for re-sequencing library construction. Two libraries with the target insert size of 300 bp were generated by the Illumina Gnomic DNA sample kit according to the manufacturer’s instruction. The quality of two libraries was controlled by qPCR. Two libraries were re-sequenced through the Illumina HiSequation 2500 at the BeiJing Berry Genomics Biotechnology Co., Ltd. (Beijing, China) to generate 125 nt paired-end short sequence reads (raw reads) for each pools.

### 4.5. Re-Sequencing Analysis

The FastQC program was used to evaluate the quality of raw reads (http://www.bioinformatics.babraham.ac.uk/projects/fastqc/).The Illumina paired-end adapters’ sequence of raw reads was removed using the FASTX toolkit program (http://hannonlab.cshl.edu/fastx_toolkit/index.html). Removal of low-quality bases (Illumina phred quality score Q < 20) [55] and ≤40 bp of reads was completed using SolexaQA software [56]. The cleaned reads from Pool (A) and Pool (B) have been submitted to the SRA database of NCBI (SRA accessions are SRP135580 and SRP135578, respectively).

The cleaned reads were aligned separately with BWA software (Burrows-Wheeler aligner) [57] to the Nipponbare reference sequence. Alignments were filtered based on the Illumina phred quality score of ≥30, corresponding to 0.1% of the error rate, to obtain the unique mapped reads. Alignment files were converted to SAM files through SAMtools [58], and applied to GATK Pipeline [59] to identify reliable SNPs based on the reference genomic sequence.

### 4.6. Calculation of ∆ (SNP Indices)

Average SNP indices of Pool (A) and Pool (B) were estimated via the sliding window method (sliding window 50 Kb; walking 10 Kb) and the ∆ (SNP indices) Manhattan plot was obtained using a custom script written in R version 3.1.1 (https://www.r-project.org/). According to the MutMap method, SNP index (A) would be 1 for the causal SNP or for closely linked SNPs and 0.5 for unlinked loci for each identified SNP in the whole genome sequence, while the SNP index (B) would be 0.333 (1/3) for the causal SNP or closely linked SNPs and 0.5 for unlinked loci. Therefore, ∆ (SNP index) would be 0.667 (2/3) for the causal or closely-linked SNPs and 0 for unlinked SNPs.

### 4.7. Restriction Endonuclease Digestion Analysis

The restriction endonuclease *Hae*II site of the target gene was identified using the primer premier 5.0 software (Premier, Ottawa, ON, Canada). Two pairs of primers were designed (W-H for WT and M-H for wb1, see Table S6) to generate 337 bp of DNA fragments by polymerase chain reaction (PCR). These PCR products were used for restriction endonuclease *Hae*II digestion. A total of 150 µL of this reaction system contained 3 µL *Hae*II (20 units per 1 µL), 15 µL NEBuffer (1×), 50 µL DNA template (2.5 µg), and 82 µL ddH_2_O. Two reaction systems were incubated at 37 °C for 15 min followed by a 2.0% agarose gel electrophoresis.

### 4.8. Vector Construction for CRISPR/Cas9-Mediated Mutation

Six novel allelic mutants were created in *Japonica* cv Nipponbare by a CRISPR/Cas-targeted genome editing tool. The pBWA(V)H_cas9i2-CRISPR/Cas9 plasmid (Figure S1) was constructed according the method described in Shan et al. (2013) [60]. To generate pBWA(V)H_cas9i2-CRISPR/Cas9 targeting vector, we used the pBWA(V)H_cas9i2 vector containing codon-optimized Cas9 driven by the 35S promoter, the OsU3 promoter and sgRNA scaffolds, as well as the Cas9 expression backbone vector. The targeting sequence primer pair was ACGTGACCTCATCAACTGGGTGG and AACGTGGCGCTGCCGAGGAACGG. The OsU3 promoter was used to drive the sgRNA expression, and the 35S promoter was used to drive the Cas9 expression. Both the OsU3::gRNA and 35S::Cas9 fragments were cloned into pBWA(V)H_cas9i2 binary vector which was introduced into *Agrobacterium* strain EHA105. Transformed calli were induced from Nipponbare seeds for *Agrobacterium*-mediated transformation as previously described [61]. The T_0_ transgenic mutant plants regenerated from hygromycin-resistant calli were examined for the presence of transgene using primer pair Cas-seq (Table S6).

## 5. Conclusions

Breeding of rice with high quality of appearance and high yield is important for rice cultivation. In this study, we isolate and characterize a candidate recessive gene *WB1* that regulates rice endosperm development using a modified MutMap method. The candidate gene *WB1* is further verified by CRISPR/Cas9 system. The *wb1* mutant, as well as six mutants mediated by CRISPR-Cas9 system, all cause a defect in the endosperm development, which lead to the higher grain chalkiness rate and degree and a significant reduction of 1000-grain weight in comparison with that of wild-type plants. Relative expression analysis of genes associated with starch synthesis by qPCR also suggests that loss of function of *WB1* leads to disorder of starch metabolism-related genes expression, resulting in the abnormal endosperm development. In particular, the modified MutMap method used in this study shows a low error rate, a relatively low cost and a high specificity, and could promote the development of rice genetics. Overall, the gene *WB1* involved in rice endosperm development affects rice quality of appearance and yield, and therefore, it can be used by rice breeders through molecular breeding to improve rice quality of appearance and yield in Green Super Rice.

## Figures and Tables

**Table 1:** Nineteen SNPs in twelve candidate genes.

Δ (SNP Index)	Accession	Location (bp)	Reference Base (WT)	Altered Base in *wb1*	Type of Mutation	Gene Annotation
0.758	*ORF1*	21550665	T	G	Missense (T to P)	Helicase conserved C-terminal domain containing protein
0.754		21550664	G	T	Missense (T to K)	
0.663		21550888	C	T	Intron mutation	
0.655		21550286	T	A	Missense (T to S)	
0.649		21551279	G	A	Intron mutation	
0.754	*ORF2*	21539737	A	G	3′-UTR mutation	Protein of unknown function DUF668 family protein
0.612		21539457	G	T	Splice region mutation	
0.743	*ORF3*	21331260	G	A	Missense (D to N)	40S ribosomal protein S10
0.734	*ORF4*	21514382	C	T	Intron mutation	Similar to H0315E07.10 protein
0.708		21513793	A	G	Intron mutation	
0.734	*ORF5*	21612944	C	A	Missense (K to N)	CENP-E-like kinetochore protein
0.663		21610862	C	A	Missense (S to I)	
0.733	*ORF6*	20423829	G	A	Missense (A to T)	Glycosyl hydrolases
0.733	*ORF7*	21795109	G	A	Missense (L to F)	Expressed protein
0.672	*ORF8*	21493980	G	A	Intron mutation	Similar to H0315E07.7 protein
0.639	*ORF9*	21897538	C	T	3′-UTR mutation	Nonsense-mediated decay UPF3
0.634	*ORF10*	21970357	C	T	5′-UTR mutation	Peptide transporter PTR2
0.631	*ORF11*	21710470	C	G	Intron mutation	Conserved hypothetical protein
0.61	*ORF12*	21734385	C	T	Nonsense (R to *)	No apical meristem protein

The asterisk indicates the stop codon.

## Supplementary Materials

Supplementary materials can be found at http://www.mdpi.com/1422-0067/19/8/2159/s1.
