# Supplementary material for: WB1, a Regulator of Endosperm Development in Rice, Is Identified by a Modified MutMap Method
Source: Int J Mol Sci. 2018 Jul 24;19(8):2159. doi: 10.3390/ijms19082159 (PMC6121324; doi:10.3390/ijms19082159)
Supplement: Supplementary file 1 [file ijms-19-02159-s001.zip › ijms-331146-SI/Supplementary.pdf]

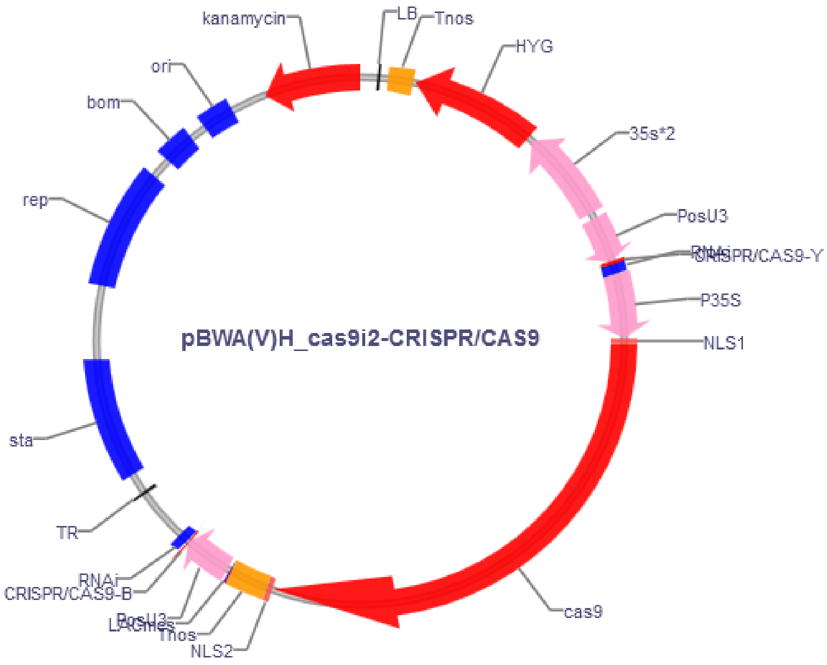

Figure S1. Vector construction for CRISPR/Cas9-mediated mutation.

Table S1. Comparison of important agronomic traits between WT and *wb1*

Trait	WT	*wb1*	*P*-Value
Plant height (cm)	52.85 ± 0.56 [1]	53.02 ± 1.08	P >0.1 [2]
No. of panicles per plant	15.90 ± 0.80	15.90 ± 0.92	P >0.1
No. of total grains/panicle	65.13 ± 1.25	51.40 ± 1.90	P <0.01
No. of filled grains/panicle	60.50 ± 1.25	36.32 ± 1.41	P <0.01
Seed-setting rate (%)	92.87% ± 0.5%	71.10% ± 2.7%	P <0.01
1000-grain weight(g)	27.67 ± 0.14	19.37 ± 0.29	P <0.01

[1] Mean ± SD. [2] Student's t-test of pairwise comparison of WT and *wb1* (**P* <0.05, ** *P* <0.01). The measurement of each trait was repeated ten times (n = 10).

Table S2. Segregation of F2 grains derived from crosses of wb1 with WT

Cross combination	F1 grains	Segregation in F2		$\chi^2_{(3:1)}$
		Wild-type	Mutant-type	
wb1×CLJ	Wild-type	820	267	0.11
wb1×ZH8015	Wild-type	745	255	0.13

Table S3. Two samples re-sequencing datasets

Samples	Conc. of DNA library (nM) [1]	Raw reads [2]	Raw length (bp) [3]	Raw bases (bp) [4]	Cleaned reads [5]	Cleaned bases (bp) [6]	Cleaned mean length (bp) [7]
Pool A	95.13	129,490,564	125	32,372,641,000	125,252,285	30,143,545,611	120.33
Pool B	93.77	124,662,017	125	31,165,504,250	120,484,878	28,985,137,170	120.29

[1] Conc. indicates the concentration of DNA libraries used for Illumina sequencing; [2] Raw reads were produced from sequencing images via base calling with Casava 1.8.; [3] The original length (125bp; PE125 mode) of Raw reads; [4] The total bases of Raw reads; [5] Cleaned reads were obtained from the treated Raw reads (see Methods); [6] The total bases of Cleaned reads; [7] The mean length of Cleaned reads.

Table S4. Result of alignments of short reads to reference sequence.

Samples	Total mapped reads [1]	Unique mapped reads [2]	Coverage (%) [3]
Pool A	125,252,285	110,119,455	87.92
Pool B	120,484,878	105,488,179	87.55

[1] Total mapped reads were the Cleaned reads; [2] Unique mapped reads were the specific reads aligned to the reference sequence in only one site, which excluded the reads aligned to the reference sequence in two or more sites; [3] Coverage was determined based on the alignments of shorts reads to the reference sequence.

Table S6. Primers used in this study

Name	Forward primer(5′→3′)	Reverse primer(5′→3′)	Purpose
W-H	CCATCGAGCCCAGCATCAG	CTCCGGTACACGTACGCCA	Enzyme digestion
M-H	CATCGAGCCCAGCATCAGG	GCTCCGGTACACGTACGCC	Enzyme digestion
Cas-seq	GCCCCTACCACGGCTGAT	CCGCCGGAAGTCCCTGCT	Sequencing
qPCR1	ACACGTACAAGGCAGGAAATA TC	TAAAACCCTACCAGAAGCCACA G	qPCR
qPCR2	CAAGTGGTGCTTCCTTCTGA	TTCCCTTTTGTCATTGGCT	qPCR
qPCR3	TTTGGTGACAGAGATGGGTA	CCTAAAGGATGGCTGGAACT	qPCR
qPCR4	CAACGGGTTCATCTTCCTGG	GAATGACCACCGATTTTAGGG	qPCR
qPCR5	GCGAGAAGACATTTGAGTTGG	ACATTATCCTCGTTTTTGGCA	qPCR
qPCR7	CAGCAGGCAGGATACCACAA	CTCCACACCAGGGGATGAC	qPCR
qPCR9	TTTGTGAATGAAAAGGGTGC	TTCGTGTTTGACTTGGGGAC	qPCR
qPCR10	GCGTAGGAGCAGTAGGTGAG	GTTCGGCGTGTCGGAGAT	qPCR
qPCR11	ATCCCTTATCTGGAGAGAAAA A	GAATGAAGAGCACCCTGAGTT	qPCR
qPCR12	CTCATTTTCTTCATTGGGGA	ACCTTTTTTCTTGTGGGACC	qPCR
Seq1-1	GGAGATTTTCCTTATGCGTTT	GTTTATTGGTGCCTCGTGCTA	Sequencing
Seq1-2	TTGGCGAAACTTATGTCATTG	ATAGTGGTCCTGTACGGCTGT	Sequencing
Seq1-3	TGATTCAACGCTCTATTTCTCTT A	ATTGTATGTTATCCCATTTGTGTG	Sequencing
Seq2	CCTTTTTATTTTTCCCCTTTTT	AGGACTACATTCTGGACCTGGT	Sequencing
Seq3	CATCTGACCACAGGGCTTC	AGGCATCTCGGATGTAGCA	Sequencing
Seq4-1	GTGTAGACAAATATGCTCTCCG	TTTGAAAAAAAGCAACACAATC	Sequencing
Seq4-2	AAAAGAAAAACGAAACGAAA G	CACAAGGCAAAATTATACCCC	Sequencing
Seq5-1	ACATTCCTTTCGTATTCCAGA	ATTGTTCCCACTTGTTCGG	Sequencing
Seq5-2	AATTTTCTTTCTTTAACTCTGCA A	TTCTTTGATTATCTGTTACCTGAA	Sequencing
Seq6-1	TCCTCTCCTCTTCTCGCTCTCAC	CACTAACTACAGTCCTCTCCCGT	Sequencing
Seq6-2	GTGGGGGTGTGCTCTGCT	GCCTTCCGGTCGTACGTT	Sequencing
Seq6-3	GAACGTACGACCGGAAGG	GGGGACGAGAAGAAAGCG	Sequencing
Seq7	TGAAAGAAATGTAGGCTAAAT GC	ACTCGGGATATGGGAAAGC	Sequencing
Seq8	AAAACTGAATGTCTGGTTAGGT G	CTTCCGATATAAATCTGTGTCGA	Sequencing
Seq9	TCAAAACAATACCACATCCCG	TGCTTCCATGAAGTGCAATCT	Sequencing
Seq10	AGTGTTGATCGAGTCGTGGG	GGCGCAAATTCATTCAGATT	Sequencing
Seq11	CTCAAGCACATAGATAAACAA CAA	CCTAGCAACATAATTTCATTAGG AT	Sequencing
Seq12	TTCTTCATTGGGGAGCCCTTT	ATGCAACTCCTGGGAGGAGATG	Sequencing
